# ^18^F-FDG PET/CT versus anatomic imaging for evaluating disease extent and clinical trial eligibility in Erdheim-Chester disease: results from 50 patients in a registry study

**DOI:** 10.1007/s00259-020-05047-8

**Published:** 2020-10-15

**Authors:** Julian Kirchner, Vaios Hatzoglou, Justin B. Buthorn, Dana Bossert, Allison M. Sigler, Anne S. Reiner, Gary A. Ulaner, Eli L. Diamond

**Affiliations:** 1 Department of Radiology, Memorial Sloan Kettering Cancer Center, New York, NY USA; 2 Department of Diagnostic and Interventional Radiology, University Dusseldorf, Medical Faculty, D-40225 Dusseldorf, Germany; 3 Department of Neurology, Memorial Sloan Kettering Cancer Center, New York, NY USA; 4 Department of Epidemiology and Biostatistics, Memorial Sloan Kettering Cancer Center, New York, NY 10022 USA; 5 Molecular Imaging and Therapy, Hoag Family Cancer Institute, Newport Beach, CA USA

**Keywords:** ^18^F-FDG PET/CT, Erdheim-Chester disease, ECD, RECIST, Modified PERCIST, Trial eligibility

## Abstract

**Objectives:**

The aim of this study was to [1] characterize distribution of Erdheim-Chester Disease (ECD) by ^18^F-FDG PET/CT and [2] determine the utility of metabolic (^18^F-FDG PET/CT) imaging versus anatomic imaging (CT or MRI) in evaluating ECD patients for clinical trial eligibility.

**Methods:**

^18^F-FDG PET/CT and corresponding CT or MRI studies for ECD patients enrolled in a prospective registry study were reviewed. Sites of disease were classified as [1] detectable by ^18^F-FDG PET only, CT/MRI only, or both and as [2] measurable by modified PERCIST (mPERCIST) only, RECIST only, or both. Descriptive analysis was performed and paired *t* test for between-group comparisons.

**Results:**

Fifty patients were included (mean age 51.5 years; range 18–70 years). Three hundred thirty-three disease sites were detected among all imaging modalities, 188 (56%) by both ^18^F-FDG PET and CT/MRI, 67 (20%) by ^18^F-FDG PET only, 75 (23%) by MRI brain only, and 3 (1%) by CT only. Of 178 disease sites measurable by mPERCIST or RECIST, 40 (22%) were measurable by both criteria, 136 (76%) by mPERCIST only, and 2 (1%) by RECIST only. On the patient level, 17 (34%) had mPERCIST and RECIST measurable disease, 30 (60%) had mPERCIST measurable disease only, and 0 had RECIST measurable disease only (*p* < 0.0001).

**Conclusion:**

Compared with anatomic imaging, ^18^F-FDG PET/CT augments evaluation of disease extent in ECD and increases identification of disease sites measurable by formal response criteria and therefore eligibility for clinical trials. Complementary organ-specific anatomic imaging offers the capacity to characterize sites of disease in greater anatomic detail.

**Trial registration:**

ClinicalTrials.gov Identifier: NCT03329274

## Introduction

Erdheim-Chester disease (ECD) is a rare and clinically heterogeneous non-Langerhans cell histiocytosis with about 1000 reported cases to date [1]. In nearly all cases, ECD is a multisystemic disease with diverse manifestations which include skeletal involvement with bone pain, exophthalmos, diabetes insipidus, xanthelasmas, interstitial lung disease, adrenal enlargement, retroperitoneal fibrosis with perirenal or ureteral obstruction, renal impairment, testis infiltration, and the involvement of the central nervous system (CNS) or cardiovascular system [2–4]. ECD diagnosis is made by a combination of clinical, radiologic, and pathologic findings; biopsy of lesional sites frequently demonstrates xanthomatous histiocytes, with the non-Langerhans immunophenotype of being CD68+, CD1a- and CD207- with admixed inflammation and fibrosis. In recent years, remarkable advances have been made in the understanding of the pathogenesis of ECD with the discovery of recurrent *BRAF*V600E and other activating mitogen-activated protein kinase (MAPK) pathway mutations in ECD tissue [5–8]. Similarly, the treatment of many ECD patients has been revolutionized by the implementation of kinase inhibitor therapies: efficacy of BRAF inhibitors vemurafenib and dabrafenib, as well as MEK inhibitors cobimetinib and trametinib, has been observed in both prospective trials [9, 10] and in meticulously documented off-trial experiences [11–14].

For several reasons, determination of the extent of ECD involvement in every patient is of pressing clinical concern. First, choice of ECD therapy is determined based on the phenotype, severity, and risk status of a patient’s disease, which depends upon the pattern of organ involvement (particularly involvement of the nervous system and heart) [15, 16]. Second, ECD patients present a tremendous burden of generalized and focal symptomatology [14, 17]. However, they frequently have concomitant diagnoses that could independently produce symptoms, such as autoimmune disease [18] and other neoplasms [19]; therefore, identification of ECD lesional sites is important to differentiate symptoms of infiltrative disease versus non-ECD symptoms. Finally, identification of ECD sites of disease may determine eligibility for clinical trials and provide evaluable target lesions for response assessment.

There is limited data about optimal imaging to characterize extent of disease in untreated ECD. Studies of small cohorts suggest that ^18^F-fluordeoxyglucose (^18^F-FDG) positron emission tomography/computed tomography (PET/CT) is informative to evaluate the extent of ECD burden [20–22]. In addition, Arnaud et al. have shown that ^18^F-FDG PET/CT has great contribution in follow-up of ECD patients receiving treatment [23]. To our knowledge, there have been no studies characterizing extent of ECD by ^18^F-FDG PET/CT in a large cohort of untreated patients. Furthermore, there has been no comparison of metabolic imaging (^18^F-FDG) versus conventional anatomic imaging (CT or MRI) with respect to identification of evaluable target lesions for clinical trial participation. The purpose of this study was two-fold. First, we sought to describe ^18^F-FDG PET/CT findings in 50 ECD patients enrolled in a prospective registry study undergoing metabolic imaging in the setting of either treatment-naive ECD or ECD that had progressed on prior chemotherapeutic or immunosuppressive therapy. Second, we sought to determine the utility of metabolic imaging versus anatomic imaging in evaluating ECD patients for measurable disease by formal response criteria, allowing for enrollment in therapeutic clinical trials.

## Material and methods

### Patients and clinical data

This single-institution study was performed in compliance with the Health Insurance Portability and Accountability Act and with the approval of the Institutional Review Board of Memorial Sloan Kettering Cancer Center (MSK). Participants in this study were enrolled in a prospective, IRB-approved ECD registry study (NCT03329274), and retrospective analysis of imaging data is performed by way of a paired retrospective analysis protocol. The registry was probed for participants with ^18^F-FDG PET/CT scans performed prior to the initiation of any kinase inhibitor therapy, in order to examine scans reflective of active disease. Demographic and clinical data collected for the registry protocol include sex, race, ethnicity, age at ECD diagnosis, presenting ECD symptoms, and ECD driver mutation (e.g., *BRAF*V600E)).

### [18]F-FDG PET/CT imaging and interpretation

At Memorial Sloan Kettering Cancer Center (MSK), image acquisition was performed on one of the several GE Healthcare Discovery PET/CT systems. Before ^18^F-FDG injection for PET/CT, patients fasted for at least 4 h. To ensure blood glucose levels below 200 mg/dl, blood samples were obtained prior injection of 370–444 Mbq (10–12 milliCurie) of ^18^F-FDG. PET/CT scans were acquired with the patient in supine position. A low-dose, attenuation correction CT scan (120 kV, 80 mA, collimation 64 × 0.625 mm, pitch 0.98, slice thickness 3.75 mm) was acquired, followed by acquisition of PET emission images. PET data were acquired for 3 min in each of up to seven bed positions (matrix size 128 × 128). Iterative image reconstruction using ordered subset expectation maximization (OSEM) was used with the following presets: two iterations, 16 subsets, 6.4 mm Gaussian transaxial filter, “heavy” *z*-axis filtering, and all manufacturers’ corrections active (CT-based attenuation, scatter, TOF, and PSF).

As the ^18^F-FDG PET/CT scan was performed to evaluate the extent of ECD burden in general and not specific for cardiovascular involvement, no cardiac-specific preparation was applied for detection of cardiovascular involvement. Subsequently, patients rested for a scheduled 60-min uptake period. A total of 15 patients had their ^18^F-FDG PET/CT examinations performed at outside institutions prior to the initiation of any ECD-specific treatment and submitted to MSKCC. ^18^F-FDG PET/CT images were reconstructed with iterative reconstruction and displayed on a PET/CT workstation (PET VCAR; GE Healthcare) as multiplanar reconstructions with multiplanar PET, CT, and PET/CT fusion images. All ^18^F-FDG PET/CT images were reviewed by a dual nuclear medicine and diagnostic radiology board certified physician (G.A.U.) with 15 years of experience in PET/CT and extensive experience with ECD radiologic assessment [9, 10, 13].

^18^F-FDG PET/CT scans were reviewed for the presence of detectable FDG-avid ECD lesions within pre-specified organ systems and, within each system, specific sites. ^18^F-FDG PET/CT of the body was reviewed for all patients, and dedicated ^18^F-FDG PET/CTs of the brain were reviewed when available. The organ systems reviewed were [1] intracranial, [2] craniofacial, [3] spinal, [4] pulmonary, [5] cardiovascular, [6] GI tract, [7], reproductive, [8] osseous, [9] skin and subcutaneous soft tissues, [10] lymph nodes and other soft tissues, and [11] other sites of disease otherwise not in the above categories.

A detectable FDG-avid lesion was defined as having visually greater standardized uptake value (SUV) than that of adjacent normal tissue, and for each lesion. the maximum SUV (SUV_max_) was measured by placing a manually drawn three-dimensional region of interest (VOI) over the lesion on attenuation-corrected PET images. A background SUV_max_ for the liver and brain was also measured for each examination and was used to determine whether each lesion was evaluable or non-evaluable by modified PERCIST (Positron Emission Response Criteria in Solid Tumors) implemented and published in ECD clinical trials [9, 24]. In accordance with previous publications, an evaluable lesion was defined as having a SUV_max_ higher than that of the liver or that of the brain for intracranial lesions [9, 24].

### CT and MRI imaging

The entire companion CT component of the ^18^F-FDG PET/CT scan was reviewed. The ^18^F-FDG PET and CT always covered identical extents of anatomic regions; dedicated CT imagings (e.g., chest/abdomen/pelvis) were reviewed, when available. The presence of ECD lesions in soft tissues, lymph nodes, and other sites was noted. Then, lesions were categorized as measurable or non-measurable according to RECIST [25]. ECD lesions considered detectable were those that demonstrated increased soft tissue at suggestive sites. For lymph nodes, an elevated short-axis diameter, irregular shape, central necrosis, and distinct contrast enhancement were criteria for involvement. Furthermore, for patients who underwent MRI brain and/or spine, a neuroradiologist experienced in the evaluation of ECD scans (V.H.) reviewed these scans to detect lesions morphologically in these two areas. Only regions that were depicted on both ^18^F-FDG PET/CT and MRI were evaluated. As with CT, lesions were again categorized as measurable or non-measurable according to RECIST.

### Statistical analysis

Statistical analysis was performed using IBM SPSS version 22 (IBM Inc., Armonk, NY, USA). The number of each type of scan analyzed (^18^F-FDG PET/CT, CT, MRI) was summarized. The number of detectable lesions, mPERCIST measurable, and RECIST measurable lesions was calculated as well as the number of individual patients with mPERCIST measurable and/or RECIST measurable lesions. Lesional SUVs were calculated per organ system as mean ± SD. Two-sided Chi-square and Fisher’s exact tests were performed to compare the number of patients with lesions measurable by mPERCIST versus RECIST versus both criteria.

## Results

### Patients and imaging studies

**Fig. 1:**
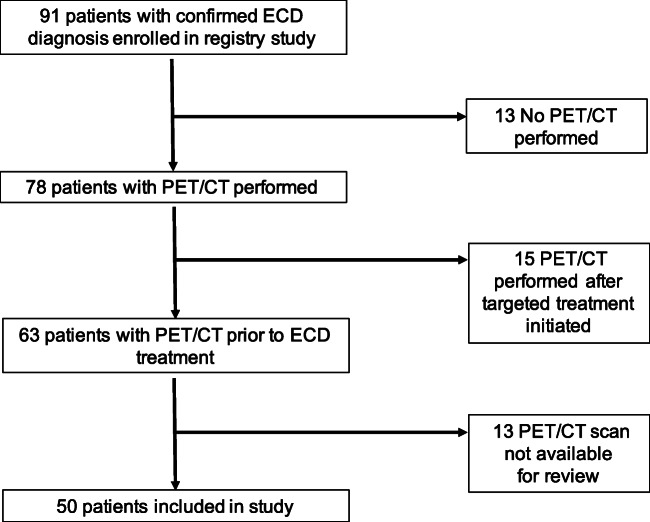
STARD diagram

A total of 50 ECD registry patients were included in this study (see STARD diagram; Fig. 1). Demographic and clinical characteristics for all patients are given in Table 1. The median age was 51.5 (range 18–70), 30 (60%) of patients were men, and 41 (82%) were Caucasian. Thirty nine (78%) had ECD only, 6 (12%) had mixed ECD and Langerhans cell histiocytosis (LCH), and 5 (10%) had mixed ECD and Rosai-Dorfman disease (RDD). Tumor mutations identified included *BRAF*V600E in 27 (54%), MAP2K1 in 6 (12%), KRAS in 3 (6%), non-V600 BRAF in 3 (6%), and others.

**Table 1:** Demographic and clinical characteristics for all patients (*N* = 50)

Characteristic	Median/*N*	Range/%	
Age	Median	Range	Comment
	51.5	18–70	
	*N*	%	
Sex			
Male	30	60%	
Female	20	40%	
Race			
Caucasian	41	82%	
African American	1	2%	
Asian	4	8%	
Not reported	4	8%	
Hispanic Ethnicity			
Yes	4	8%	
No	45	90%	
Not reported	1	2%	
ECD diagnosis			
ECD only	39	78%	
Mixed ECD/Langerhans cell histiocytosis	6	12%	
Mixed ECD/Rosai-Dorfman disease	5	10%	
ECD symptoms			
Constitutional	30	60%	
Musculoskeletal pain	24	48%	
Visual or ocular	13	26%	
Gastrointestinal	12	24%	
Skin or subcutaneous	15	30%	
Cardiac	10	20%	
Respiratory	8	16%	
Renal or urologic	14	28%	
Neurologic or neuro-endocrine	30	60%	
Psychiatric	15	30%	
Endocrine	29	58%	
ECOG status			
0	24	48%	
1	13	26%	
2	6	12%	
Unknown	7	14%	
ECD mutation			
BRAFV600E	27	54%	
BRAF non-V00E	3	6%	BRAF-PICALM fusion, ANP32A-BRAF fusion, BRAF p.N486_P490indel)
KRAS	3	6%	*KRAS*G12S, *KRAS*L19F, *KRAS*R149G
MAP2K1	6	12%	*MAP2K1* p.P105_I107indel, *MAP2K1*C121S, *MAP2K1*P124Q, *MAP2K1*p.I103-A106del, *MAP2K1*G128D, *MAP2K1*F53L
MAP2K2	1	2%	*MAP2K2*Y143H
ARAF	2	4%	*ARAF*S214A, *ARAF*S214F
RAF1	1	2%	
ALK	1	2%	KIF5B-ALK fusion
Multiple mutations	1	2%	*KRAS*G12R, *ARAF*P216A
No mutation identified	5	10%	

In 39 patients, a whole-body ^18^F-FDG PET/CT including the brain and legs was performed, and in 11 patients, the examination ranged from skull base to thighs (limited whole-body field of view). In 37 cases, ^18^F-FDG PET/CT was performed prior any ECD treatment was given; and in 13 cases the patients had progressive disease following chemotherapy or immunosuppressive treatments but had not received kinase inhibitor therapy. Dedicated CT of the chest, abdomen, and pelvis is performed in 15 (30%) of patients, and MRI of the brain is performed in 41 (82%) patients (Table 2).

**Table 2:** PET, CT, and MRI images analyzed for all patients (*N* = 50)

Imaging modality		*N*	%
Body Imaging		50	100%
Head-to-toe PET/CT		39	78%
Skull-to-thigh PET/CT		11	22%
Contrast-enhanced CT (in addition to PET/CT)		15	30%
Neuroimaging		41	82%
MRI brain			

### Disease distribution

**Fig. 2:**
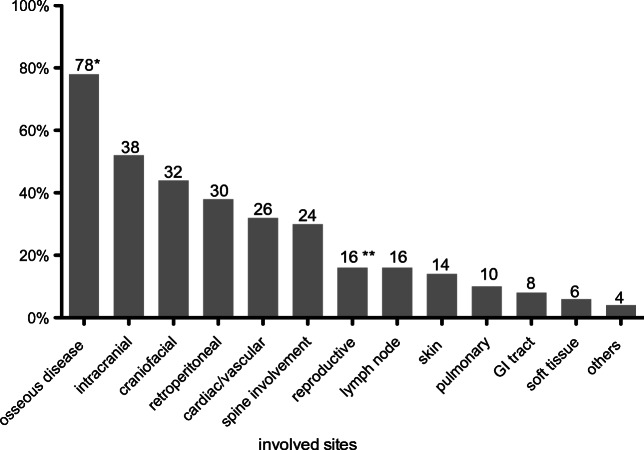
Frequencies of radiographic findings in patients by ECD in ^18^F-FDG PET/CT and/or MRI. *78% out of entire cohort of 50 and 97% out of patients who had head to toe PET/CT. **16% out of entire cohort of 50 and 27% out of male patients

Among all imaging studies performed (^18^F-FDG PET, CT, and MRI), a total of 333 sites of disease involvement are detected (Tables 3 and 4). Based on the ^18^F-FDG PET/CT studies, the most commonly involved site is the skeletal system with detectable lesions in 39 of 50 patients (78%) (see Table 3, Fig. 2). However, among the 39 patients with imaging of the legs, 38 (97%) had disease involvement in the legs. Retroperitoneal involvement was identified in 19 patients (38%), with perinephric soft tissue (“hairy kidney”) in 15 patients (30%). Cardiac and aortic disease was the fourth most common site of disease with 16 affected patients (32%), mainly due to peri-aortic infiltration of the thoracic (24%) or the abdominal aorta (14%), whereas in 6 of the patients (12%), the thoracic and abdominal aorta were involved. The reproductive organs and lymph nodes were involved in 8 patients (16%) and the skin in 7 patients (14%); of note, only men had detectable involvement of reproductive organs (testes) (i.e., 27% of men). Pulmonary (parenchymal or pleural) disease occurred in 5 patients (10%) and gastrointestinal involvement in 4 patients (8%). Additional soft tissue involvement was seen in 3 patients (6%). Other sites of involvement include the liver and paraspinal musculature in one patient each (Table 3).

**Table 3:** Organ systems and sites of disease involvement, detectable on PET, CT, or both for 50 patients

	PET or CT	PET and CT	PET only	CT only
	*N* total (% patients)	*N* total (% patients)	*N* total (% patients)	*N* total (% patients)
Osseous	39 (78%)			
Clavicle	4 (8%)	2 (4%)	2 (4%)	0 (0%)
Scapula	5 (10%)	4 (8%)	1 (2%)	0 (0%)
Sternum	5 (10%)	4 (8%)	1 (2%)	0 (0%)
Arms	17 (34%)	17 (34%)	0 (0%)	0 (0%)
Pelvis	17 (34%)	11 (22%)	5 (10%)	1 (2%)
Legs	38 (76%)*	36 (72%)	1 (2%)	1 (2%)
Retroperitoneal	19 (38%)			
Adrenal	9 (18%)	9 (18%)	0 (0%)	0 (0%)
Perinephric soft tissue (hairy kidney)	15 (30%)	14 (28%)	1 (2%)	0 (0%)
Paranephric soft tissue	10 (20%)	8 (16%)	1 (2%)	1 (2%)
Hydronephrosis	4 (8%)	4 (8%)	0 (0%)	0 (0%)
Pancreas	1 (2%)	1 (2%)	0 (0%)	0 (0%)
Cardiac/vascular	16 (32%)			
Right atrium/septum	8 (16%)	4 (8%)	4 (8%)	0 (0%)
Pericardium	1 (2%)	1 (2%)	0 (0%)	0 (0%)
Peri-aortic infiltration of				
- Thoracic aorta	12 (24%)	6 (12%)	6 (12%)	0 (0%)
- Abdominal aorta	7 (14%)	2 (4%)	5 (10%)	0 (0%)
Coated aorta	4 (8%)	2 (4%)	2 (4%)	0 (0%)
Spine involvement	15 (30%)			
Spinal cord	3 (6%)	0 (0%)	3 (6%)	0 (0%)
Vertebral bodies	10 (20%)	9 (18%)	1 (2%)	0 (0%)
Epidural space	6 (12%)	1 (2%)	5 (10%)	0 (0%)
Others	2 (4%)	2 (4%)	0 (0%)	0 (0%)
Intracranial	13 (26%)			
Cerebral hemispheres	6 (12%)	3 (6%)	3 (6%)	0 (0%)
Cerebellum	5 (10%)	1 (2%)	4 (8%)	0 (0%)
Dura	3 (6%)	0 (0%)	3 (6%)	0 (0%)
Hypothalamus/pituitary/infundibulum	5 (10%)	4 (8%)	1 (2%)	0 (0%)
Pseudo-meningioma	1 (2%)	0 (0%)	1 (2%)	0 (0%)
Brainstem	7 (14%)	3 (6%)	4 (8%)	0 (0%)
Craniofacial	12 (24%)			
Orbital	5 (10%)	5 (10%)	0 (0%)	0 (0%)
Paranasal sinus	9 (18%)	9 (0%)	0 (0%)	0 (0%)
Reproductive organs**	8 (16%)			
Testes	8 (16%)**	1 (2%)	7 (9%)	0 (0%)
Lymph nodes	8 (16%)	6 (12%)	2 (4%)	0 (0%)
Skin or subcutaneous	7 (14%)	7 (14%)	0 (0%)	0 (0%)
Pulmonary	5 (10%)			
Pleural	2 (4%)	2 (4%)	0 (0%)	0 (0%)
Parenchymal mass	2 (4%)	2 (4%)	0 (0%)	0 (0%)
Larynx	1 (2%)	0 (0%)	1 (2%)	0 (0%)
GI tract	4 (8%)			
Upper tract	1 (2%)	1 (2%)	0 (0%)	0 (0%)
Omentum/peritoneum	3 (6%)	3 (6%)	0 (0%)	0 (0%)
Soft tissue	3 (6%)	3 (6%)	0 (0%)	0 (0%)
Others	2 (4%)	1 (2%)	1 (2%)	0 (0%)
Total lesions	258	188	67	3

*76% out of entire cohort of 50 and 97% out of patients who had head to toe PET/CT

**16% out of entire cohort of 50 and 27% out of male patients

**Table 4:** Cranial sites of disease involvement, detectable on PET, MRI, or both for 41 patients

	PET or MRI	PET and MRI	PET only	MRI only
	*N* total (% patients)	*N* total (% patients)	*N* total (% patients)	*N* total (% patients)
Intracranial	24 (59%)			
Cerebral hemispheres	11 (26%)	3 (7%)	1 (2%)	7 (17%)
Cerebellum	11 (26%)	3 (7%)	1 (2%)	7 (17%)
Dura	7 (17%)	3 (7%)	0 (0%)	4 (10%)
Hypothalamus/pituitary/infundibulum	13 (32%)	4 (10%)	2 (4%)	7 (17%)
Pseudo-meningioma	2 (4%)	0 (0%)	1 (2%)	1 (2%)
Brainstem	13 (32%)	5 (12%)	1 (2%)	7 (17%)
Corpus callosum	1 (2%)	0 (0%)	0 (0%)	1 (2%)
Craniofacial	22 (54%)			
Orbital	10 (24%)	4 (10%)	0 (0%)	6 (14%)
Paranasal sinus	11 (24%)	8 (20%)	0 (0%)	3 (7%)
Lacrimal gland	3 (7%)	0 (0%)	0 (0%)	3 (7%)
Skull base or mandible	12 (29%)	0 (0%)	0 (0%)	12 (29%)
Calvarium involvment	17 (41%)	0 (0%)	0 (0%)	17 (41%)
Total lesions	111	30	6	75

Considering neurologic (cranial) regions evaluated by either ^18^F-FDG PET/CT or MRI in the 41 patients who underwent additional brain MRI, disease involvement was detected in 29 (58%) of the patients. Here, the most common site is intracranial disease with 26 patients (52%), followed by craniofacial with 22 patients (44%) disease and spine involvement with 15 patients (30%) (Table 4).

**Fig. 3:**
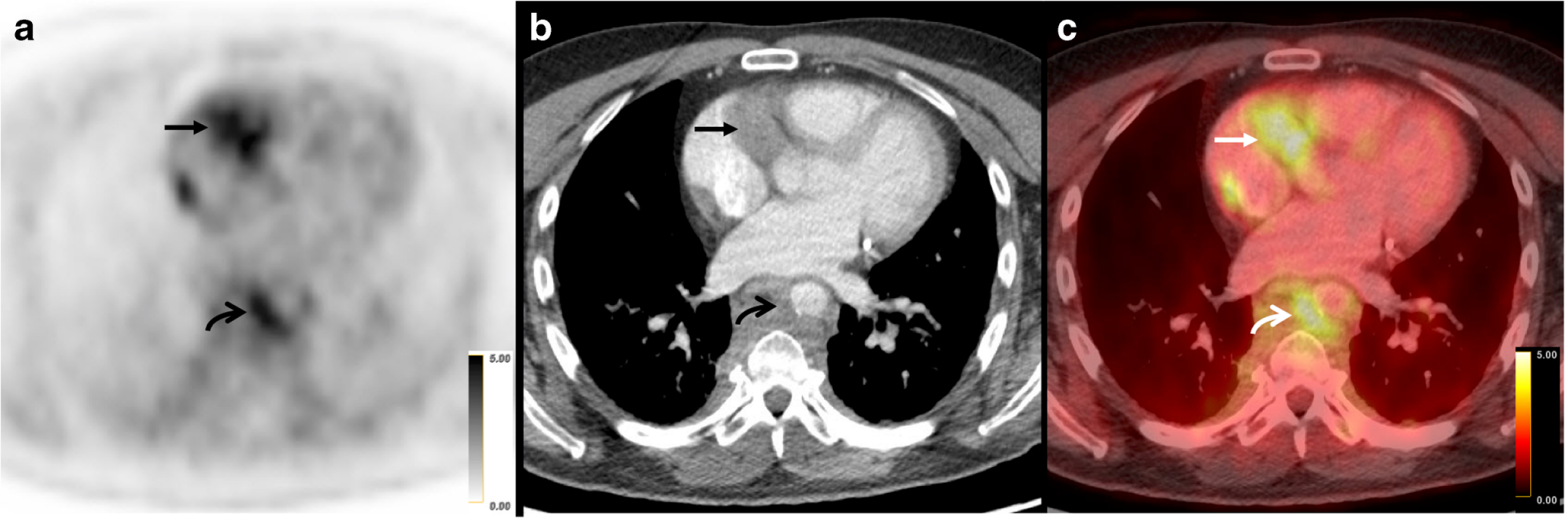
ECD detectable and measurable on both FDG PET and CT in a 53-year-old male. (**a**) Axial FDG PET, (**b**) axial CT, and (**c**) axial-fused FDG PET/CT through the level of the heart demonstrate FDG-avid cardiac soft tissue with a SUV_max_ of 4.2 (arrows), as well as FDG-avid peri-aortic soft tissue with a SUV_max_ of 4.0 (curved arrows)

Of the 333 total sites of disease, 188 (56%) lesions could be detected by both ^18^F-FDG PET and CT/MRI (Fig. 3). Seventy-five (23%) lesions could only be detected by MRI specifically, and all of these lesions were located in the brain. Sixty-five lesions (20%) were only detectable by ^18^F-FDG PET, and 3 lesions were only detectable by CT (1%).

Additionally, we compared contrast-enhanced PET/CT versus non-contrast PET/CT for retroperitoneal and cardiovascular regions, which are the sites of disease anticipated to differ by the presence or absence of contrast administration. For cardiovascular sites of disease, contrast-enhanced PET/CT detected lesions in 5 of 15 (33%) patients versus 11 of 35 (31%) for non-contrast PET/CT. For retroperitoneal sites of disease, contrast-enhanced PET/CT detected lesions in 7 of 15 (46%) patients versus 12 of 35 (34%) for non-contrast PET/CT.

### Modified PERCIST and RECIST-measurable lesions

**Fig. 4:**
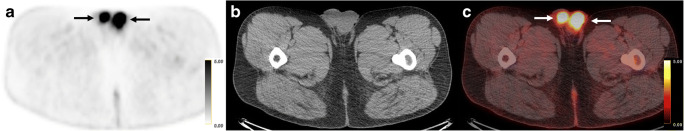
ECD detectable on FDG PET, but not on CT, in a 41-year-old male. (**a**) Axial FDG PET, (**b**) axial non-contrast CT, and (**c**) axial-fused FDG PET/CT through the level of the testes demonstrate FDG-avid bilateral testis ECD with an SUVmax of 12.0 (arrows). While the disease is detectable on FDG PET and measurable by mPERCIST, the testes are of normal size and without focal lesion on CT

Forty seven out of the 50 patients (94%) had one or more ECD lesion that was trial evaluable by ^18^F-FDG PET (mPERCIST) or CT/MRI (RECIST; Table 4). All 47 patients had measurable lesions by mPERCIST (94%; 47 of 50), while 17 (34%; 17 of 50) had measurable lesions by RECIST (Fig. 4). Three patients did not have lesions evaluable by mPERCIST or RECIST.

**Fig. 5:**
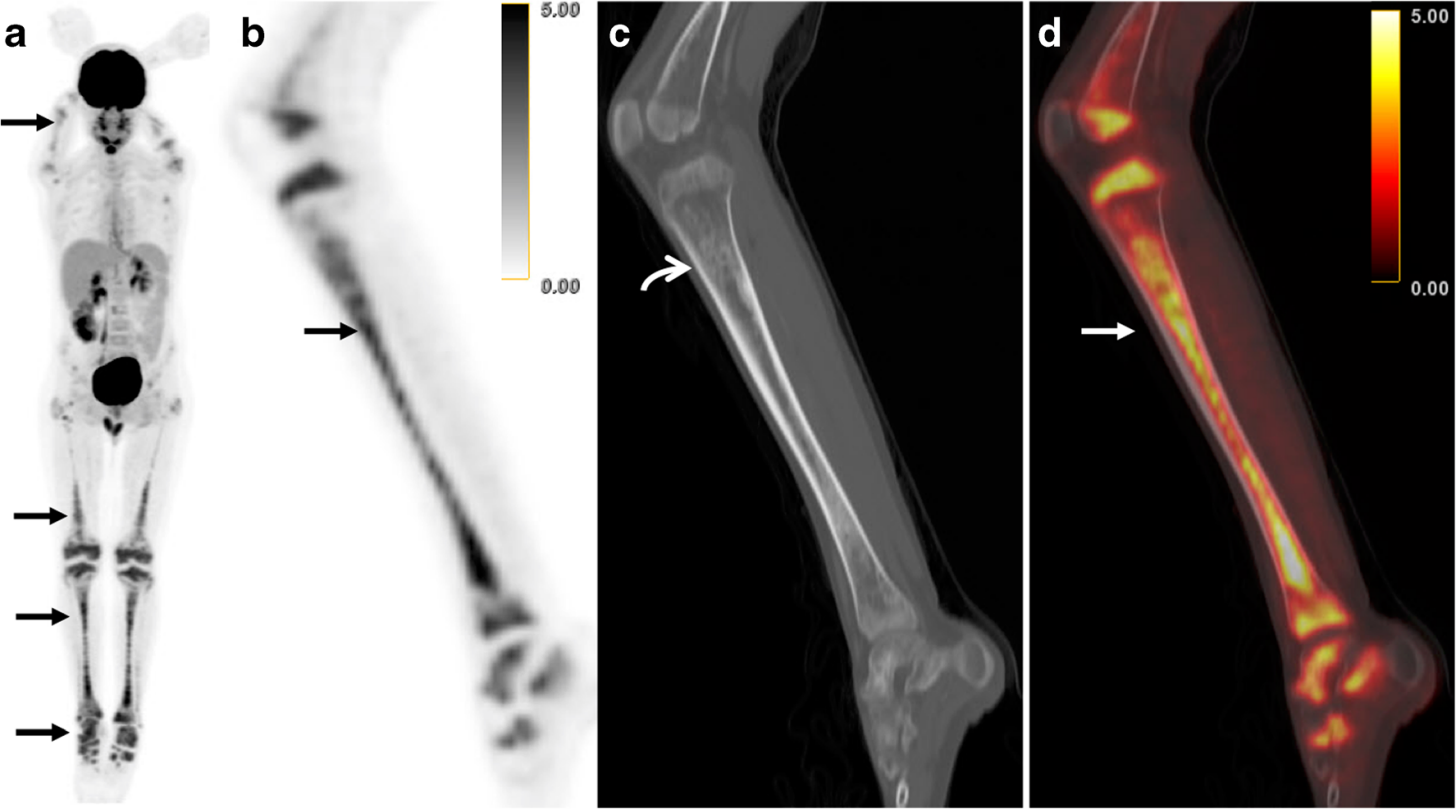
ECD detectable by both FDG PET and CT, but measurable only by mPERCIST, in an 18-year-old male. **a** FDG MIP demonstrates multi-focal abnormal FDG-avidity, greatest in the lower extremities (arrow). (**b**) Sagittal FDG PET, (**c**) sagittal CT, and (**d**) sagittal-fused FDG PET/CT centered on the left tibia demonstrate FDG-avid osseous ECD with an SUV_max_ of 5.2 (arrows). The CT demonstrates sclerotic correlates (curved arrow) that are detectable on CT, but not measurable by RECIST

Out of the 333 lesions, 178 were trial evaluable by mPERCIST or RECIST. Of these, 136 (76%) sites were only measurable by mPERCIST, 40 sites (22%) were measurable by both mPERCIST and RECIST, and just two sites (1%) were only measurable by RECIST. The most significant discrepancy is with respect to bone lesions; here 33 sites are only trial-eligible by mPERCIST compared and no lesion by RECIST (Fig. 5). For exact distribution, please see Table 5.

**Table 5:** Identification of measurable disease by modified PERCIST and RECIST on a lesion-based analysis and on a patient-based analysis

Lesion-based			
	mPERCIST and RECIST	mPERCIST only	RECIST only
	*N* total (% patients)	*N* total (% patients)	*N* total (% patients)
Osseous	0	33	0
Scapula	0	1	0
Arms	0	3	0
Pelvis	0	6	0
Legs	0	23	0
Retroperitoneal	7	15	2
Adrenal	0	1	0
Perinephric soft tissue	1	10	0
Pararenal soft tissue	6	3	2
Pancreas	0	1	0
Cardiac/vascular	9	19	0
Right atrium	3	5	0
Pericardium	1	0	0
Thoracic aorta	3	9	0
Abdominal aorta	2	5	0
Spine involvement	1	17	0
Spinal cord	0	3	0
Vertebral bodies	0	9	0
Dural/epidural space	0	4	0
Others	1	1	0
Intracranial	3	23	0
Cerebral hemispheres	2	4	0
Cerebellum	0	4	0
Dura	0	3	0
Hypothalamus	0	6	0
Brainstem	1	6	0
Craniofacial	5	10	0
Orbital	5	0	0
Paranasal sinus	0	8	0
Others	0	2	0
Reproductive organs	1	7	0
Lymph nodes	3	5	0
Skin or subcutaneous	4	1	0
Pulmonary	4	1	0
Pleura	2	0	0
Parenchyma	1	1	0
Others	1	0	0
GI tract	0	4	0
Upper tact	0	1	0
Peritoneum/omentum	0	3	0
Soft tissue	2	1	0
Others	1	0	0
Total	40	136	2
Total lesions	178		
Patient-based (*N* = 50)			
mPERCIST or RECIST	mPERCIST and RECIST	mPERCIST only	RECIST only
*N* total (% patients)	*N* total (% patients)	*N* total (% patients)	*N* total (% patients)
47 (94)	17 (34)	30 (60)	0

The higher rate of clinical trial eligible patients with ^18^F-FDG PET/CT was statistically significant for comparing mPERCIST and/or RECIST measurable patients to RECIST only evaluable patients (47 vs 0; *p* < 0.0001). Furthermore, there were significantly more patients that are only mPERCIST measurable than mPERCIST and RECIST evaluable patients (30 vs 17; *p* < 0.0001).

### ^18^F-FDG PET/CT values (SUV_max_)

The SUV_max_ of the involved sites showed a wide range. The highest uptake was seen in intracranial lesions with a mean SUV_max_ of 21.9 (SD 11.2). The mean SUV_max_ of the brain background activity was 12.5. Highest uptake of body lesions was seen in pulmonary lesions and lymph nodes with an uptake about 11 and lowest in spine involvement and cardiac/vascular with uptake about 5. The mean SUV_max_ of the liver was 2.9. Please see Table 6 for mean uptake of all involved sites.

**Table 6:** Average SUV_max_ values for sites of involvement that could be followed with subsequent PET scans

Localization	SUV_max_ ± StdDev	Range
Intracranial	21.9 ± 11.2	6.4–42.9
Pulmonary	11.3 ± 9.4	3.0–25.6
Lymph nodes or soft tissue	11.0 ± 12.9	3.2–50.0
Retroproductive organs	8.5 ± 3.6	5.2–16.4
GI tract	8.2 ± 5.6	2.2–8.9
Retroperitoneal	7.9 ± 7.4	2.2–8.9
Craniofacial	7.1 ± 4.1	2.9–19.1
Skin or subcutaneous	6.4 ± 3.1	3.0–11.4
Osseous disease	6.2 ± 4.2	2.3–18.8
Spine involvement	5.6 ± 2.8	2.3–12.4
Cardiac/vascular	4.7 ± 2.0	2.3–11.7

## Discussion

In this study, we present ^18^F-FDG PET/CT and CT/MRI data for 50 patients with ECD, an ultra-rare hematopoietic neoplasm, enrolled in a prospective registry study. We examined metabolic and anatomic imaging performed at the time of diagnosis, or in the setting of active previously treated disease for the presence of ECD lesions and their evaluability by clinical trial response criteria, specifically modified PERCIST and RECIST. We found a substantially greater number of lesions to be both identifiable (215 vs 91 for body) and formally evaluable (176 vs 42) by metabolic versus anatomic criteria. The sites of disease involvement most discrepant with respect to detectability and evaluability by metabolic and anatomic criteria were osseous and spinal structures.

With respect to frequency of sites of ECD involvement, our findings confirm results of two other prospective cohorts [26, 27] and one systematic review of ECD findings [28] with some informative differences. As is known, bone lesions are the most common site of disease in ECD. We found lesions in the legs to be nearly invariably present (38 of 39 with PET/CT of the legs), similar to the study by Estrada-Veras et al. [27] in which all participants underwent nuclear imaging (in the case of this study, bone scan) of the legs. The relatively less frequent presence of leg lesions in other studies may reflect the fact that not all patients in these other cohorts underwent nuclear imaging of the legs. We identified cardiac or cardiovascular involvement in 32% of our patients, compared with 51% in the Pitié-Salpêtrière series, and we specifically detected substantially fewer instances of right atrial (16% versus 36%) and pericardial (2% versus 29%) lesions. We would hypothesize the reason for this to be that our patients were not evaluated with cardiac MRI which would be the most sensitive modality to detect such lesions. Conversely, we identified intracranial lesions in 52% of our cohort, more than what has been reported in other series, and we would suggest that this is the result of frequent MRI evaluation of our patients. In terms of frequency of symptoms reported by our patients, there is no other cohort, to our knowledge, collecting prospective patient-reported data about ECD symptomatology. Constitutional symptoms, pain, and neurologic symptoms were highly frequent in our patients (60%, 48%, and 60%, respectively).

Our study evaluating SUV values in ECD lesions had similar findings to the one existing study on this topic by Arnaud et al. [23]. Intracranial lesions demonstrated the greatest FDG-avidity of all lesions, with mean SUV_max_ of 21.9. This is likely due the high brain background FDG-avidity (mean brain background SUV_max_ 12.5), which increases the threshold for detecting FDG-avid intracranial lesions.

Arnaud et al. evaluated ^18^F-FDG PET/CT in the initial and follow-up evaluation of ECD patients. Specifically, they examined 65 scans among 31 patients with ECD, 23 of whom were untreated at the time of ^18^F-FDG PET/CT. ^18^F-FDG PET/CT was analyzed in its sensitivity and specificity to anatomic imaging, CT or MRI for different organ systems, assuming the latter was the gold standard (100% sensitive and specific) for identifying ECD lesions. The study found that the sensitivity of [18]F-FDG PET/CT with respect to CT/MRI varied widely among organ systems, but that [18]F-FDG PET/CT was nearly 100% specific. Our study was different from this study in that [1] we did not assume that either modality was the gold standard, and also [2] we compared PET to CT outside of the nervous system where the prior study used MRI in other instances. Overall, we do not find our results at odds with this study, although our study highlights the possibility that [18]F-FDG PET/CT can detect lesions not seen on CT and therefore is of diagnostic value in the initial ECD evaluation. Our study also focused on the specific question of evaluability by clinical trial response criteria, suggesting that [18]F-FDG PET/CT is particularly valuable for this purpose.

One of the main findings of our study is that [18]F-FDG PET/CT is superior to CT with respect to defining measurable disease in patients with ECD, allowing eligibility into clinical trials. While FDG PET/CT has not been as important in solid tumor clinical trials as for lymphoma, this may be evolving. For example, a recent study [29] comparing [18]F-FDG PET/CT response criteria and RECIST in the context of a molecular basket trial found that 14 of 81 patients had tumors that were evaluable by [18]F-FDG PET alone, highlighting the value of [18]F-FDG PET in expanding eligibility for clinical trials. One important explanation for the marked discrepancy between mPERCIST and RECIST evaluability in our patient population is the fact that osseous lesions are not evaluable by RECIST. Histiocytic neoplasms, and ECD in particular, often present with osseous lesions, and therefore [18],F-FDG PET/CT-derived response criteria may be particularly well suited for defining measurable disease in ECD. In a recent trial of cobimetinib for histiocytic neoplasms, an overall response rate of 89% was observed by mPERCIST, compared with an overall response rate of 57% by RECIST, suggesting that [18]F-FDG PET/CT more readily detects response to treatment. The broad recommendation for implementation of [18]F-FDG PET/CT for response assessment in ECD [30] and other histiocytosis [31] reflects a belief among experts that metabolic assessment is useful to gauge response to treatment and that favorable metabolic response reflects a clinical benefit from treatment, but this has not been proven in ECD.

There are limitations to this study. Most importantly, we compared [18]F-FDG PET/CT with whole-body, low-density, non-contrast CT in 35 of 50 patients, and we acknowledge that this may have limited our capacity to detect lesions by anatomic imaging. We would offer the explanation that a dedicated contrast-enhanced CT was likely done at some point in the diagnostic trajectory of many patients; however, there is often a long delay to diagnosis in ECD from the time of initial evaluation, Therefore, by the time the [18]F-FDG PET/CT was performed for the specific purpose of ECD staging, the dedicated CT would not be repeated. Our sample size does not allow for statistical comparison of non-contrast versus contrast enhanced CT, but our study suggests that contrast-enhanced CT may have a greater capacity to detect lesions, especially at retroperitoneal regions. Second, in some patients, we compared [18]F-FDG PET/CT with CT, rather than MRI, for organs that are better suited for MRI evaluation, such as the spine and cardiovascular structures. Therefore, we interpret with caution the degree of discrepancy between [18]F-FDG PET/CT-detected and CT-detected lesions in these areas. Nonetheless, our data suggest the strong capacity for [18]F-FDG PET/CT to detect ECD lesions across organ systems. Finally, we do not wish to conflate *detection* of ECD lesions, or establishing their evaluability by trial response criteria, with *characterization* of lesions’ precise location and infiltration of structures. We would propose, based on our data, that [18]F-FDG PET/CT is particularly useful in identifying sites of disease in ECD and that organ-specific CT or MRI can be used to further characterize sites of disease with respect to size, specific location, and granular features.

In summary, we have presented [18]F-FDG PET/CT finding from 50 patients enrolled in a prospective ECD registry, highlighting the utility of [18]F-FDG PET/CT in identifying sites of histiocytic disease, particularly disease sites that are measurable by formal response criteria, allowing patients to meet inclusion criteria for enrollment onto therapeutic clinical trials. Longitudinal investigation would shed light on the relationship between changes in metabolic and anatomic aspects of disease and changes in clinical parameters such as organ function and disease-related symptoms.
